# A Common Sense Pivot Tooth

**Published:** 1885-08

**Authors:** L. C. Adams

**Affiliations:** Dayton, O.


ARTICLE V.

### A COMMON SENSE PIVOT TOOTH.

BY DR. L. C. ADAMS, DAYTON, O.

As all readers of the dental periodicals know, there are many ways of setting artificial crowns on crownless roots. The writer has tried a number of them, and has had satisfactory success with some, and total failures with others. Whether the failures resulted from defects in the plan, or from inability to properly manipulate, let the reader judge. And right here I wish to call attention to the fact that many roots supposed to be worthless, can be made useful by setting artificial crowns on them.

A case in my practice illustrates the efficiency of the principle: A gentleman called to have the roots of three front teeth extracted, and corresponding teeth inserted on a plate. Seeing that the roots were healthy, I advised him to have crowns set on them, and thus avoid a plate. He consented, and the work was done, and now after a lapse of eight months they are doing good service, and have given full satisfaction. The teeth had been broken off from these roots many years before.

The mode of making and applying the tooth with which I have been most successful, is very simple; and any dentist that can use a blowpipe, and flow hard solder, can adapt it with complete success.

First prepare the root, as for the old method of setting a pivot tooth with the antediluvian wooden peg. Make a hole in the root about a quarter of an inch in depth. A good drill for making the hole may be made by taking a fissure bur drill, three thirty-seconds of an inch in diameter, and grinding to the desired shape. Enlarge the hole slightly at the upper end, then make a tube that will snugly fill the hole without being to tight, leaving the tube projecting a very little. Use platinum, or silver. Then make a plate to fit the prepared root, having the same diameter as the root. Make a hole in the plate to correspond with that in the root, and just large enough to admit the end of the tube. Fit the two nicely together, and fasten with wax, and then remove and invest in sand and plaster, equal parts, and solder the plate and tube together, and also the seam of the tube, if not previously done, allowing no solder to flow into the tube. After this take a plain plate or rubber tooth, and fit it to the root, seeing that when thus fitted, it properly antagonizes. Grind it back well so as to give free access to the hole in the tube, then back-line the tooth and fit it to the root, with the plate and tube in place, and then fasten as before, and remove and solder together. After this, warm the tooth, and place it on some gutta-percha softened by heat, and insert into place on the root, and hold fimly until it has become cool, and then remove and trim off any surplus gutta-percha. By using the gutta-percha thus, a water-tight joint is secured. Dry the root with warm air, and fill the hole in it with well mixed cement, and press the tooth firmly to its place on the root, and with suitable instruments, pack the cement into the tube until it is full. After it has hardened, drill a portion of the cement away, and fill that portion of the tube with amalgam, mixed as dry as it will work; and this completes the process.

This mode of adapting the tooth was first brought to my notice by Dr. C. Bradley, of Dayton, Ohio.

